# First molecular evidence of *Rickettsia* spp. in *Triatoma rubrofasciata*: implications for vector ecology and zoonotic transmission

**DOI:** 10.1186/s13071-026-07489-9

**Published:** 2026-06-13

**Authors:** Binglian Qin, Wenting Wei, Lili Tang, Peichao Deng, Xiaoquan Liu, Ling Li, Qingan Zhou, Yuanlin Hou, Huitong Pan, Jiaming Liang, Xiaoyin Fu, Shanshan He, Yanwen Li, Dengyu Liu, Yunliang Shi

**Affiliations:** 1 Parasitology Department, School of Basic Medical Sciences, Guangxi Medical University, No.22 Shuangyong Road, Qingxiu District, Nanning, 530021 China; 2 The First People’s Hospital of Nanning, Nanning, People’s Republic of China; 3 Animal Disease Prevention and Control Center of Guangxi Zhuang Autonomous Region, Nanning, People’s Republic of China; 4 Key Laboratory of Basic Research on Regional Diseases (Guangxi Medical University), Education Department of Guangxi Zhuang Autonomous Region, Nanning, People’s Republic of China; 5 University Engineering Research Center of Advanced Technologies in Medical and Biological Intelligent Manufacturing of Guangxi, Nanning, People’s Republic of China; 6 Lianyungang Customs Integrated Technology Center, Lianyungang, People’s Republic of China

**Keywords:** *Triatoma rubrofasciata*, *Rickettsia felis*, Southern China, Molecular detection, Molecular characteristics, One Health

## Abstract

**Background:**

*Rickettsia* is an emerging global pathogen with incompletely understood transmission cycles. While triatomine bugs are established vectors for *Trypanosoma cruzi*, their potential role in the ecology of *Rickettsia* spp. remains unexplored. This study investigated the presence and genetic characteristics of *Rickettsia* spp. in *Triatoma rubrofasciata* and sympatric hosts in southern China.

**Methods:**

A total of 362 *T. rubrofasciata* specimens including the fecal samples, head tissues and gut were screened for *Rickettsia* DNA using nested PCR targeting six genes (*groEL*, *rrs*, *gltA*, *17-kDa*, *ompA*, and *ompB*). In addition, 64 fleas, 136 ticks, and 69 wild rodents were tested. Positive amplicons were sequenced and subjected to phylogenetic analysis.

**Results:**

*Rickettsia* DNA was detected in eight (2.2%, 8/362) *T. rubrofasciata* specimens, with *Rickettsia felis* specifically identified in four of these positive samples; the species of the remaining four could not be determined. The bacterium was detected across multiple anatomical sites, including the head (which contains the salivary glands), the gut, and fecal samples. Surveillance of sympatric hosts revealed a markedly higher prevalence in fleas (57.8%) and wild rats (4.3%), whereas ticks harbored a distinct agent related to *Candidatus* Rickettsia jingxinensis (29.4%). Genomic analyses indicated high conservation of key loci among *R. felis* strains across hosts, with a stable *ompA* gene variation distinguishing vector- and rodent-derived lineages. Phylogenetically, all *R. felis* sequences formed a distinct clade separate from tick-associated rickettsiae.

**Conclusions:**

This study confirms for the first time the presence of *Rickettsia* in *T. rubrofasciata*, including *R. felis* and *Rickettsia* sp. The *R. felis* was found co-circulating in fleas and rodents, while ticks harbored a distinct *Rickettsia*, indicating separate transmission cycles in sympatric hosts. Genetic divergence in *ompA* further suggests host-adaptive evolution between vector- and rodent-associated strains. These findings suggest that *T. rubrofasciata* may serve as a previously unrecognized interface in the epidemiology of flea-borne spotted fever in southern China. They also highlight the expanded vector range of *Rickettsia* and underscoring the ecological complexity of its transmission, calling for broader surveillance of nontraditional vectors in endemic regions.

**Graphical Abstract:**

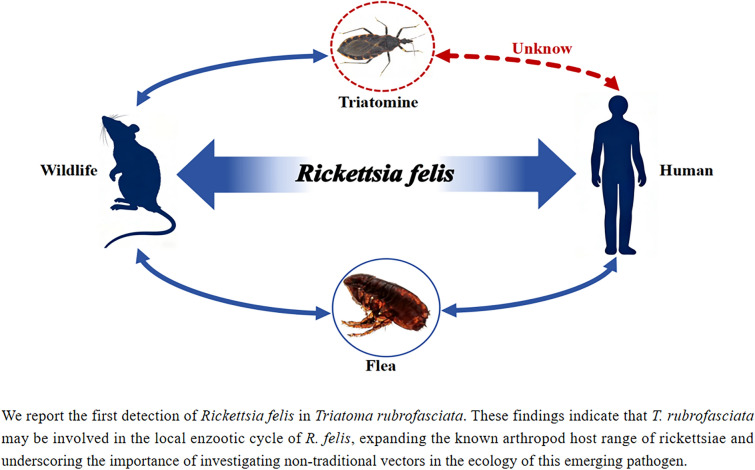

**Supplementary Information:**

The online version contains supplementary material available at 10.1186/s13071-026-07489-9.

## Background

*Rickettsia* spp. are obligate intracellular, Gram-negative bacteria that primarily infect eukaryotic cells and are maintained in nature through complex cycles involving hematophagous arthropods and vertebrate reservoirs [1]. These pathogens are classified into four major phylogenetic groups: the ancestral group (AG), spotted fever group (SFG), typhus group (TG), and transitional group (TRG) [2]. Among them, several species are recognized as significant human pathogens, including *Rickettsia prowazekii* (epidemic typhus), *Rickettsia typhi* (murine typhus), *Rickettsia rickettsii* (Rocky Mountain spotted fever), *Rickettsia felis* (flea-borne spotted fever), and *Rickettsia japonica* (Japanese spotted fever) [3]. In addition, at least 20 other *Rickettsia* species have been associated with human infections worldwide [1, 4].

The global burden of rickettsial diseases remains substantial. In the USA, cases of tick-borne spotted fever rickettsiosis (SFR) increased by 46% from 2016 to 2017 [5]; among these, Rocky Mountain spotted fever carries a case-fatality rate of 5–10%. Without treatment, this rate can reach up to 20% [5, 6]. Scrub typhus, caused by *Orientia tsutsugamushi*, threatens an estimated 1 billion people globally and affects over 1 million annually [7], with a reported case fatality rate of 1.5% in a rural cohort study in southern India, where baseline IgG sero-negativity was associated with increased susceptibility to severe disease and diabetes mellitus conferred a 2.4-fold increased risk of severe infection [8]. In China, rickettsioses represent an emerging public health concern. Between 2005 and 2024, scrub typhus was the most prevalent vector-borne disease in China, with a cumulative incidence of 23.27 cases per 100,000 population, and a reported mortality rate of 0.03% [9]. In addition, spotted fever rickettsiosis, though less common, has also resulted in fatal outcomes, including a confirmed death in Hubei Province in 2021 attributed to Japanese spotted fever [10]. Over the past decades, at least eight SFG *Rickettsia* species have been identified in ticks across multiple Chinese provinces, four of which have been reported to infect humans [11], underscoring the country’s diverse rickettsial ecology.

Transmission of *Rickettsia* occurs predominantly through bites or fecal contamination by infected arthropods, with ticks, lice, and fleas serving as primary vectors [12]. Ticks are the main vectors for SFG rickettsiae such as *R. rickettsii*, *R. sibirica*, and *R. conorii* [1, 4]. Body lice transmit *R. prowazekii* via contaminated feces [13], while fleas, particularly the cat flea, *Ctenocephalides felis* (Bouché, 1835), are the only known biological vectors and reservoirs for *R. felis* [14]. Rodents and other small mammals act as key reservoir hosts, maintaining enzootic transmission cycles that occasionally spill over into human populations. Notably, triatomine bugs—best known as vectors of *Trypanosoma cruzi* (Chagas, 1909) in the Americas—have been surveyed for *Rickettsia* infection, but none were detected [15].

*Triatoma rubrofasciata* (De Geer, 1773) is distributed worldwide. Surveys indicate that it is widely distributed across Guangxi Zhuang Autonomous Region, Hainan, Guangdong, Fujian, and Taiwan Provinces [16–20], with a trend of further spread [21]. This insect thrives in intradomiciliary and peridomestic environments, including wooden bed boards, wall crevices, woodpiles, and livestock and poultry shelters, where it maintains close contact with humans and domestic animals [17]. These insects frequently feed on humans, domestic animals, and wildlife [22]. Given their broad host range and blood feeding behavior, *T. rubrofasciata* has been hypothesized to harbor or transmit pathogens other than *T. cruzi* [23, 24]. However, despite the widespread circulation of *Rickettsia* in both arthropod vectors and mammalian hosts in China, the role of *T. rubrofasciata* in rickettsial ecology has never been investigated.

This study aimed to investigate the potential role of *T. rubrofasciata* in the enzootic cycle of *Rickettsia* in southern China. We specifically sought to: (1) determine the presence and prevalence of *Rickettsia* DNA in field-collected *T. rubrofasciata*, (2) genetically characterize and phylogenetically position any detected rickettsiae, and (3) assess the potential integration of triatomine bugs into the local transmission network of *R. felis* by comparing with sympatric fleas, ticks, and rodents. Our findings provide the first molecular evidence of *R. felis* in *T. rubrofasciata*, thereby expanding the known vector range of this pathogen and highlighting the importance of surveillance beyond traditional vectors in rickettsial ecology.

## Methods

### Ethical considerations

All procedures involving experimental animals were conducted in strict accordance with the Guidelines for the Care and Use of Laboratory Animals issued by the Ministry of Science and Technology of China. The study protocol was reviewed and approved by the Institutional Animal Care and Use Committee of Guangxi Medical University (approval no. 82260413). According to the local natural resources bureau, the scientific trapping of pest rodent species in residential areas falls outside the scope of a specific collection permit.

### Sample collection and species identification

Field collections were conducted between 2023 and 2025 across southern China. *T. rubrofasciata* specimens (*n* = 362) were collected from Guangxi Zhuang Autonomous Region and Hainan Province. Specimens were obtained through two approaches: active field surveys conducted by the research team, and capture by local residents, who subsequently submitted the specimens to us for a fee. All live specimens were transported to the laboratory and maintained in semi-closed cubic containers (12 cm × 8 cm × 6 cm). The habitat of the collected triatomine bugs was recorded. Habitat types were classified as intradomiciliary (intra; inside houses, e.g., wooden bed boards and wall crevices) or peridomiciliary (peri; surrounding structures, e.g., debris piles under eaves, woodpiles, and poultry shelters).

Fleas (*n* = 64) were collected in 2024 from stray cats and dogs housed at an animal rescue center in Nanning, Guangxi; fleas recovered from the same host were pooled as a single sample. Ticks (*n* = 136) were removed from cattle and pet dogs in five cities of Guangxi during 2023–2025, with ticks from each individual host grouped together. Wild rodents (*n* = 69) were trapped in 2024–2025 using snap traps and live traps in residential areas and pigeon farms in Nanning and peridomestic zones near Yizhou, Hechi. All arthropod and rodent specimens were identified to species level based on morphological criteria and confirmed by molecular methods.

### Sample processing and DNA extraction

Specimens of *T. rubrofasciata* were collected and maintained under controlled laboratory conditions and fed on specific-pathogen-free (SPF) Kunming mice. During culture, fresh fecal samples were collected from each specimen following blood meals. For individuals that did not defecate spontaneously, gentle intestinal abdominal compression was applied to induce defecation. The triatomines were surface-sterilized in a biosafety cabinet by sequential immersion in 75% ethanol (3 × 3 min), followed by three rinses in 1× phosphate-buffered saline (PBS; 3 × 3 min), and then air-dried. Appendages (antennae, legs, mouthparts, and wings) were removed. Subsequently, under aseptic conditions, the head (including salivary glands) and gut were dissected separately using sterile instruments. All samples from the same individual, including serial fecal samples, head tissues, and gut, were assigned a common identification code and stored individually at −80 °C for subsequent DNA extraction.

For fleas, three individuals per host-derived pool were selected, surface-cleaned using the same protocol as above, and processed whole. For ticks, the dorsal cuticle was carefully removed with a sterile scalpel, and internal tissues-including the midgut and salivary glands-were harvested. Rodents were humanely euthanized following anesthesia with tribromoethanol (2.5%, 0.8–1.2 mL per 100 g body weight) via cervical dislocation. Approximately 10 mg of cardiac tissue was aseptically excised for analysis.

Total genomic DNA was extracted from all samples using the TIANamp Genomic DNA Kit (Tiangen Biotech, Beijing, China; cat. no. DP304-03), according to the manufacturer’s instructions. Extracted DNA was quantified spectrophotometrically and stored at −80 °C until use.

### Molecular detection and sequencing of *Rickettsia* spp.

All DNA samples were screened by nested PCR targeting the *Rickettsia* heat-shock protein gene (*groEL*), *16S rRNA* (*rrs*), citrate synthase (*gltA*), 17-kDa antigen (*17-kDa*), outer membrane protein A (*ompA*), and outer membrane protein B (*ompB*) (Table 1), genes commonly used for species-level discrimination within the genus *Rickettsia*. Each 25-µL PCR reaction contained: 12.5 µL of 2× PCR Premix (Takara Bio, Japan), 1 µL each of forward and reverse primers (10 µM), 2 µL of template DNA, and 8.5 µL of nuclease-free water. Negative controls (nuclease-free water instead of DNA) were included in every run. For the second-round amplification, 1 µL of the first-round product served as template. To prevent contamination in nested PCR, strict physical separation of pre- and post-amplification steps was performed, and no-template negative controls (nuclease-free water instead of DNA) were included in each run to monitor potential cross-contamination.

**Table 1:** Primer sets used for *Rickettsia* detection

Group	Gene	Primer name	Sequence	Amplification conditions	Size (bp)	Reference
SFG	*groEL*	Gro1	AAGAAGGACGTGATAAC	94 °C, 5 min; 39 cycles: 94 °C, 40 s, 45 °C, 40 s, 72 °C, 40 s; 72 °C, 4 min	649	[25]
		Gro2	ACTTCACGTAGCACC			
		SF1	GATAGAAGAAAAGCAATGATG	94 °C, 5 min; 40 cycles: 94 °C, 35 s, 56 °C, 35 s, 72 °C, 35 s; 72 °C, 4 min	217	
		SR2	CAGCTATTTGAGATTTAATTTG			
TG		TF1	ATATATCACAGTACTTTGCAAC		364	
		TR2	GTTCCTAACTTAGATGTATCAT			
SFG	*ompB*	ompB OF	GTAACCGGAAGTAATCGTTTCGTAA	95 °C, 5 min; 35 cycles: 95 °C, 15 s, 54 °C, 15 s, 72 °C, 30 s; 72 °C, 3 min	512	[26]
		ompB OR	GCTTTATAACCAGCTAAACCACC			
		ompB IF	GTTTAATACGTGCTGCTAACCAA	95 °C, 5 min; 35 cycles: 95 °C, 15 s, 56 °C, 15 s, 72 °C, 30 s; 72 °C, 3 min	425	
		ompB S/T IR	GGTTTGGCCCATATACCATAAG			
TG		ompB TG IF	AAGATCCTTCTGATGTTGCAACA		249	
		ompB S/T IR	GGTTTGGCCCATATACCATAAG			
SFG	*rrs*	Ric-F	YTACGGAATAACTTTTAGAAA	94 °C, 2 min; 30 cycles: 94 °C, 30 s, 50 °C, 1 min, 72 °C, 2 min; 72 °C, 10 min	900	[27]
		Ric-R1	CATGATGACTTGACRTCGT			
		Ric-R2	CATCTCACGACACGAGCTG			
	*gltA*	gltA1	GATTGCTTTACTTACGACCC	95 °C, 5 min; 35 cycles: 95 °C, 30 s, 52 °C, 30 s, 72 °C, 1 min; 72 °C, 4 min	1087	[28]
		gltA2	TGCATTTCTTTCCATTGTGC			
		gltA3	TATAGACGGTGATAAAGGAATC	95 °C, 5 min; 35 cycles: 95 °C, 30 s, 53 °C, 30 s, 72 °C, 1 min; 72 °C, 4 min	667	
		gltA4	CAGAACTACCGATTTCTTTAAGC			
	*17-kDa*	17KDa-1W	GCTTTACAAAATTCTAAAAACCATATA	95 °C, 5 min; 35 cycles: 95 °C, 60 s, 58 °C, 60 s, 72 °C, 60 s; 72 °C, 5 min	550	[29]
		17KDa-2W	TGTCTATCAATTCACAACTTGCCGTT			
		17KDa-3N	GCTCTTGCAACTTCTATGTT	95 °C, 5 min; 35 cycles: 95 °C, 30 s, 61 °C, 30 s, 72 °C, 30 s; 72 °C, 5 min	434	
		17KDa-4N	CATTGTTCGTCAGGTTGGCG			
	*ompA*	Rr190k.70F	TGGCGAATATTTCTCCAAAA	94 °C, 5 min; 35 cycles: 94 °C, 40 s, 50 °C, 50 s, 72 °C, 1 min; 72 °C, 7 min	650	[30]
		Rr190k.720pR	TGGCGAATATTTCTCCAAAA			
		Rr190k.70F	TGGCGAATATTTCTCCAAAA	94 °C, 5 min; 35 cycles: 94 °C, 40 s, 48 °C, 60 s, 72 °C, 2 min; 72 °C, 7 min	532	
		Rr190k.602nR	AGTGCAGCATTCGCTCCCCCT			

Amplified products were resolved by 1.2% agarose gel electrophoresis and visualized under UV light. Positive amplicons were purified using the Agarose Gel DNA Extraction Kit (Meiji Biotechnology, Guangzhou, China; cat. no. D2111-02). Purified DNA was submitted to Shanghai Sangon Biotech Co., Ltd. (Guangzhou Branch) for bidirectional Sanger sequencing.

### Sequence analysis and phylogenetic reconstruction

Obtained sequences were compared against the National Center for Biotechnology Information (NCBI) nucleotide database using BLASTn (https://blast.ncbi.nlm.nih.gov/Blast.cgi) to identify closest matches and select representative reference sequences from diverse geographic regions and host origins. Multiple sequence alignments were performed using DNAMAN (version 6.0) for the *17-kDa*, *ompA*, and *ompB* coding regions, incorporating homologous sequences retrieved from GenBank (Table S1).

Phylogenetic relationships were inferred using both maximum-likelihood (ML) and neighbor-joining (NJ) methods implemented in MEGA11 (version 11.0.13). For ML analysis, the general time reversible model with gamma distribution and invariant sites (GTR + G + I) was used, while evolutionary distances for NJ analysis were computed using the Kimura 2-parameter model. Bootstrap support was assessed with 1000 replicates for both methods. Representative *Rickettsia* sequences from multiple vector species were selected as references (Table S2).

## Results

### Species identification of collected specimens

**Fig. 1:**
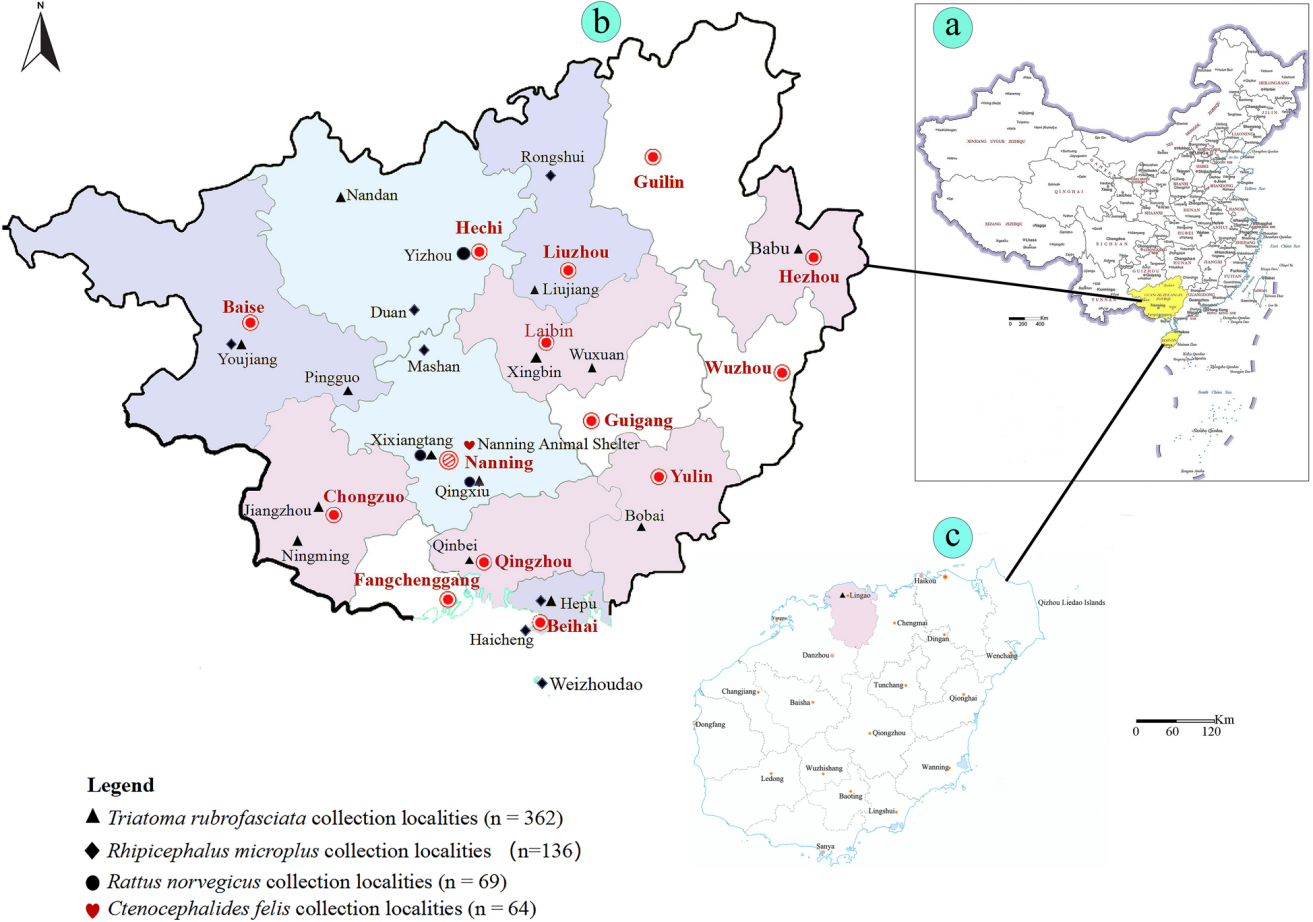
**Collection sites and sample sizes of *Triatoma rubrofasciata* , *Ctenocephalides felis* , *Rattus norvegicus* and *Rhipicephalus microplus* in Guangxi Zhuang Autonomous Region and Hainan Province, 2023–2025.** (a) Location of Guangxi and Hainan within China. (b) Guangxi Zhuang Autonomous Region. (c) Hainan Province.

**Fig. 2:**
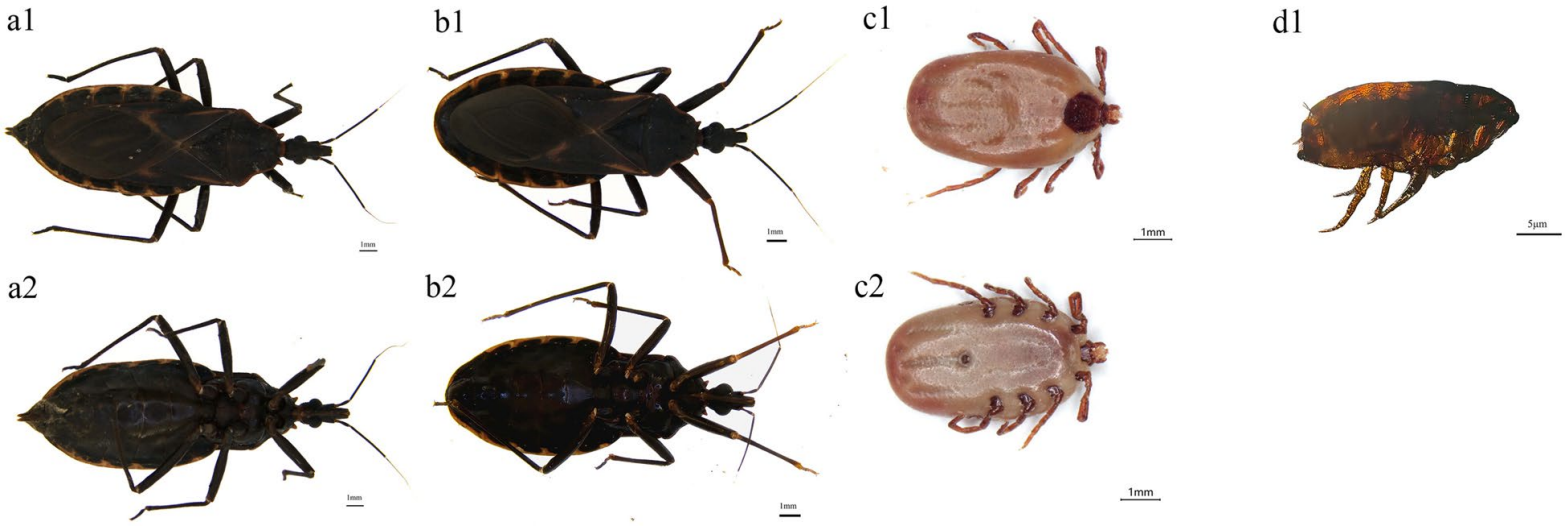
**Morphological identification of *Triatoma rubrofasciata* , *Rhipicephalus microplus* and *Ctenocephalides felis* collected in selected areas of Guangxi and Hainan, China, 2023–2025.** *T. rubrofasciata* female: (a1) dorsal view and (a2) ventral view; T. rubrofasciata male: (b1) dorsal view and (b2) ventral view; R. microplus : (c1) dorsal view and (c2) ventral view; C. felis : (d1) lateral view.

A total of 362 triatomine bugs were collected from 11 localities: 10 in Guangxi Zhuang Autonomous Region (Nanning, Chongzuo, Yulin, Beihai, Laibin, Baise, Hechi, Liuzhou, Hezhou. Qinzhou) and 1 in Hainan Province (Lingao County) (Fig. 1; Table S3). All specimens were morphologically identified as *T. rubrofasciata* (Fig. 2) using a Nikon SMZ745T stereomicroscope and diagnostic keys described by Xiao [31], supplemented by developmental-stage illustrations from the US Centers for Disease Control and Prevention (CDC) website (https://www.cdc.gov/chagas/hcp/species/).

In addition, 136 ticks were collected from cattle and pet dogs in five Guangxi cities (Nanning, Beihai, Hechi, Liuzhou, and Baise) (Fig. 1). Morphological examination (Fig. 2) combined with *16S rRNA* gene sequencing confirmed all ticks as *Rhipicephalus microplus* (Canestrini, 1888) [32]. Overall, 64 fleas were obtained from stray cats and dogs at an animal shelter in Nanning (Fig. 1); both morphological traits (Fig. 2) and mitochondrial cytochrome *c* oxidase subunit I (*COI*) gene analysis identified them as *C. felis* [33]. Wild rodents (*n* = 69) captured in Yizhou (Hechi) and peridomestic areas of Nanning (Qingxiu and Xixiangtang districts) (Fig. 1) were identified as *Rattus norvegicus* (Berkenhout, 1769) on the basis of morphology and cytochrome *b* (*Cytb*) gene sequencing [34].

### Detection and molecular characterization of *Rickettsia* in *T. rubrofasciata*

Nested PCR targeting six genetic loci (*groEL*, *rrs*, *gltA*, *17-kDa*, *ompA*, and *ompB*) was employed to screen 362 *T. rubrofasciata* individuals for *Rickettsia*. From each specimen, feces were collected, and the insect was then dissected to obtain head tissues and gut separately. Consequently, 362 head samples, 362 gut samples, and 362 fecal samples were independently analyzed by PCR. Eight specimens (2.2%) yielded amplification of at least one target gene and were considered *Rickettsia* positive. These included seven from Guangxi (specimen codes: GxTrRi-4, -10, -11, -24, -26, -57, and -87) and one from Hainan (HnTrRi-5). Among the eight triatomines that tested positive in fecal samples, two (GxTrRi-4 and GxTrRi-11) showed concurrent detection in head tissues and gut; four (GxTrRi-24, -26, -57, and -87) were positive in gut, with head tissues testing negative; one (GxTrRi-10) was positive in feces and head tissues, with gut testing negative; and one (HnTrRi-5) was positive in feces, with head tissues and gut testing negative (Table 2; Table S4).

**Table 2:** *Rickettsia* species identification in *Triatoma rubrofasciata*, *Ctenocephalides felis*, *Rattus norvegicus*, and *Rhipicephalus microplus*

Sample species	No. examined	No. positive (%)	Identified species
Triatomines	362	8 (2.2%)	*Rickettsia felis* (*n* = 4)
			*Rickettsia* sp. (*n* = 4)
Fleas	64	37 (57.8%)	*Rickettsia felis* (*n* = 37)
Rats	69	3 (4.3%)	*Rickettsia felis* (*n* = 1)
			*Rickettsia* sp. (*n* = 2)
Ticks	136	40 (29.4%)	*Candidatus* Rickettsia jingxinensis (*n* = 40)
Total	631	88 (13.9%)	

BLASTn analysis revealed that the amplified sequences from all eight positive isolates exhibited the highest nucleotide identity to *R. felis*: *groEL* (98.87–99.42%; GenBank MT019639.1), *rrs* (99.77–100%; MT003287.1), *gltA* (100%; KF242471.1), *17-kDa* (99.73–100%; MK242042.1), *ompA* (99.56–100%; KY913639.1), and *ompB* (99.21–100%; PP820517.1/PQ151995.1) (Table S4). Based on established taxonomic criteria for *Rickettsia* species delineation [35]—which require ≥ 99.8% sequence identity in the *rrs* gene,  ≥ 99.9% in *gltA*,  ≥ 98.8% in *ompA*, and ≥ 99.2% in *ompB*—four isolates (GxTrRi-4, GxTrRi-24, GxTrRi-57, and HnTrRi-5) that yielded positive amplification for three or more of these loci met the threshold for classification as *R. felis*. In contrast, the remaining four isolates (GxTrRi-10, GxTrRi-11, GxTrRi-26, and GxTrRi-87), which were detected in only a single gene target, could not be assigned to a named species and were therefore conservatively designated as *Rickettsia* sp. (Table 2; Table S4).

### Prevalence and diversity of *Rickettsia* in fleas, ticks, and rodents

Parallel screening of 64 cat fleas, 136 ticks, and 69 wild rodents revealed markedly higher *Rickettsia* prevalence in fleas (57.8%, 37/64) and ticks (29.4%, 40/136) compared with triatomines, with a lower but detectable rate in rodents (4.3%, 3/69) (Table 2). In fleas, almost all six target genes were amplified. Sequencing of representative amplicons (*n* = 59) confirmed all as *R. felis*, with 96.28–100% identity to reference strains (Table 2; Table S4). In contrast, tick-derived sequences (*n* = 59 from ten representative samples) clustered consistently with *Candidatus* Rickettsia jingxinensis, showing 99–100% identity across *rrs*, *gltA*, *17-kDa*, *ompA*, and *ompB*. Only the *groEL* locus showed slightly lower similarity (96.11–100%), consistent with known genetic variation in this gene among novel rickettsial lineages. Rodent cardiac tissue samples produced nine *Rickettsia*-positive amplicons derived from three individuals, showing sequence identities of 97.48–100% to *R. felis* across the targeted loci (*rrs*, *gltA*, *17-kDa*, *ompA*, and *ompB*). On the basis of the established taxonomic criteria for *Rickettsia* [35], isolate GxRaRi-66 can be classified as *R. felis*, whereas isolates GxRaRi-33 and GxRaRi-36 were closely related to *R. felis* but could not be definitively assigned to this species (Table 2; Table S4).

### Comparative sequence analysis of *R. felis* across hosts

**Fig. 3:**
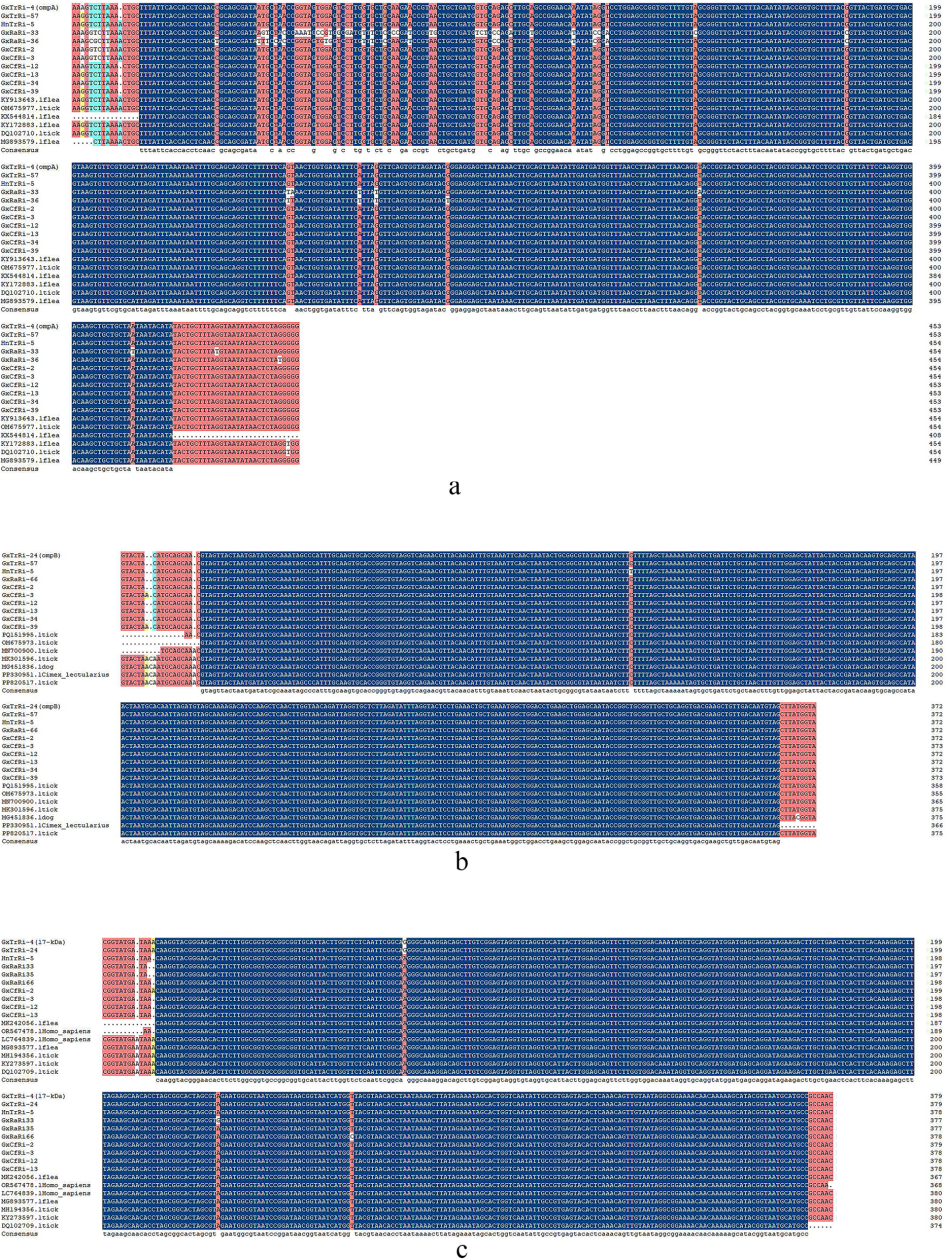
**Multiple sequence alignment of *ompA* , *ompB* and *17-kDa* genes of Rickettsia felis derived from different host sources.** (a) *ompA* gene. (b) *ompB* gene. (c) *17-kDa* gene. The alignment includes sequences obtained from *Triatoma rubrofasciata* (specimen codes *ompA* : GxTrRi-4, -57, HnTiRi-5; *ompB* : GxTrRi-24, -57, HnTiRi-5; *17-kDa* : GxTrRi-4, -24, HnTiRi-5), *Ctenocephalides felis* ( *ompA* : GxCfRi-3, -12, -13, -34, -39; *ompB* : GxCfRi-2, -3, -12, -13, -34, -39; *17-kDa* : GxCfRi-2, -3, -12, -13), and *Rattus norvegicus* ( *ompA* :GxRaRi-33, -36; *ompB* : GxRaRi-66; *17-kDa* : GxRaRi-33, -36, -66), together with corresponding sequences from other host species retrieved from NCBI (accession numbers in Additional file 1: Table S1). The variant sites in the sequence alignment were highlighted.

Multiple sequence alignments of *ompA*, *ompB*, and *17-kDa* genes from *R. felis* isolates recovered from *T. rubrofasciata*, *C. felis,* and *R. norvegicus* revealed high conservation in the *ompA* gene, which showed complete identity between *R. felis* from *T. rubrofasciata* and *C. felis*. However, two rodent-derived strains (GxRaRi-33 and GxRaRi-36) harbored a distinct haplotype characterized by 12 shared nucleotide substitutions relative to flea- and triatomine-associated strains (positions 34, 39, 58, 60, 63, 69, 88–89, 108, 173, 240, 257, and 261) (Fig. 3a). The observed genetic variation suggests that *R. felis* may have undergone host-adaptive evolution in both vector and animal hosts. By contrast, *ompB* and *17-kDa* showed high degree of sequence consensus among hosts (Fig. 3b, c).

### Phylogenetic relationships of detected *Rickettsia* strains

**Fig. 4:**
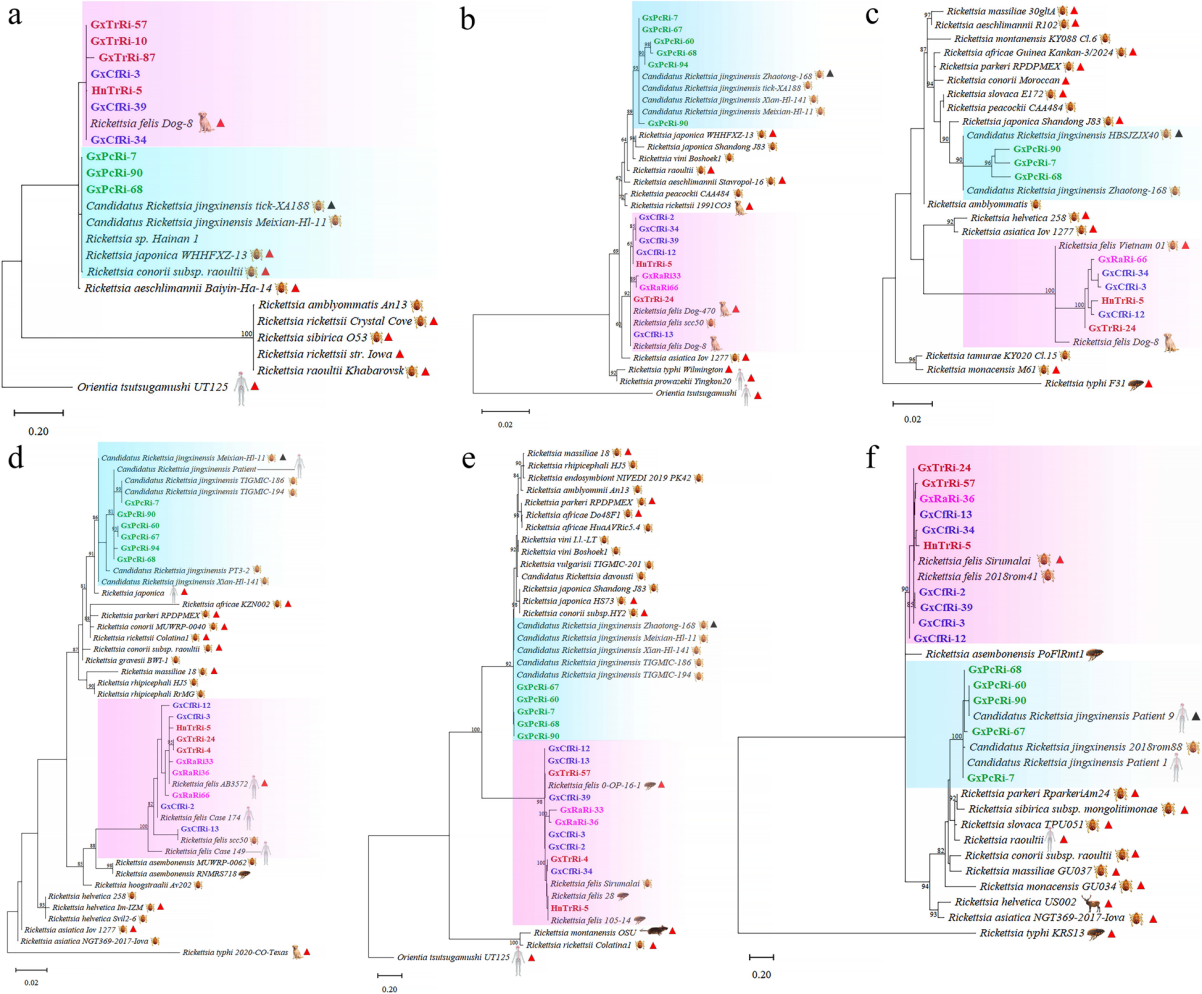
**Phylogenetic trees of *Rickettsia* spp. inferred from the *groEL , rrs , gltA , 17-kDa , ompA ,* and *ompB* gene sequences.** (a) *groEL* . (b) *rrs* . (c) *gltA* . (d) *17-kDa* . (e) *ompA* . (f) *ompB* . Sequences generated in this study from *Triatoma rubrofasciata*, *Ctenocephalides felis*, *Rattus norvegicus*, and *Rhipicephalus microplus* are highlighted in red, blue, purple, and green, respectively; corresponding GenBank accession numbers are listed in Additional file 2: Table S2. Host species of reference sequences are denoted by pictograms. *Rickettsia* taxa documented as human pathogens are indicated by red triangles, whereas those detected in humans but of unresolved pathogenicity are marked with black triangles. Trees were constructed using the maximum likelihood method in MEGA11 with 1,000 bootstrap replicates. In all six loci, *T. rubrofasciata* - and *C. felis* -derived sequences grouped within the *R. felis* clade, while *R. microplus*-derived sequences formed the *Candidatus* R. jingxinensis clade. *R. norvegicus*-derived sequences were included in four loci (*rrs*, *gltA*, *17-kDa*, *ompA*) and clustered with *R. felis* .

Phylogenetic trees were reconstructed for each of the six *Rickettsia*-specific loci (*groEL*, *rrs*, *gltA*, *17-kDa*, *ompA*, and *ompB*) using ML and NJ methods, with *R. typhi* and *O. tsutsugamushi* serving as outgroups. Both methods produced congruent topologies across all loci. The ML trees are shown in the main text (Fig. 4), whereas the NJ trees are provided in Additional file 5 (Fig. S1). Across all loci, two distinct phylogenetic lineages emerged: one corresponding to *R. felis* and the other to *Candidatus* R. jingxinensis (Fig. 4). In the *groEL* phylogeny, the four *T. rubrofasciata*-derived and three flea-derived sequences were clustered tightly with dog-associated *R. felis* strains, while tick-derived sequences formed a separate clade with *Candidatus* R. jingxinensis (Fig. 4a). Similarly, *rrs* sequences from two triatomines (GenBank: PX755671.1), five fleas (GenBank: PX755674.1, PX755675.1), and two rodents (PX755672.1, PX755673.1) grouped with *R. felis* reference strains from dog and tick, whereas six tick sequences aligned exclusively with *Candidatus* R. jingxinensis from ticks (Fig. 4b). In the *gltA* tree, two isolates from *T. rubrofasciata*, three flea isolates, and one rodent co-clustered with reference *R. felis* strains from dog and tick, distinct from the tick-associated *Candidatus* R. jingxinensis group (Fig. 4c).

The *17-kDa* phylogeny further reinforced this pattern, with triatomine, flea, and rodent sequences forming a monophyletic *R. felis* clade that clustered with the *R. felis* isolates from human patients in Thailand and Vietnam, indicating a close genetic relationship with human-pathogenic strains. The *Candidatus* R. jingxinensis from tick in this study cluster with *Candidatus* R. jingxinensis from humans and ticks (Fig. 4d). *ompA* and *ompB*—key markers for species delineation within the spotted fever group—showed that *R. felis* sequences from *T. rubrofasciata* and *C. felis* grouped together with high bootstrap support, while all tick-derived sequences consistently associated with *Candidatus* R. jingxinensis (Fig. 4e, f). Overall, these multi-locus phylogenetic results indicate that *R. felis* derived from triatomine bugs is closely related to human-, flea-, dog-, and tick-associated strains, suggesting that *R. felis* circulates in a multi-host transmission system involving fleas, rodents, and—as newly identified in this study—*T. rubrofasciata*.

## Discussion

The classical vectors encompass ixodid ticks (e.g., *Dermacentor* and *Rhipicephalus* spp.) [36, 37], chigger mites [38], human body lice [39], and fleas such as *Ctenocephalides* and *Xenopsylla* [40]. These vectors transmit rickettsiae either through direct inoculation during feeding or via contamination of bite wounds or mucous membranes with infected feces. A previous study in French Guiana screened seven triatomine species (*Rhodnius prolixus* Stål, 1859; *Rhodnius pictipes* Stål, 1872; *Rhodnius robustus* Larrousse, 1927; *Triatoma infestans* (Klug, 1834); *Panstrongylus geniculatus* (Latreille, 1811); *Panstrongylus rufotuberculatus* (Champion, 1899); and *Eratyrus mucronatus* (Stål, 1859) for *Rickettsia* spp., but found no evidence of infection [15]. Therefore, it remains unknown whether *Triatoma* species can carry and transmit *Rickettsia*. In this study, we present the first molecular evidence that *T. rubrofasciata*, a synanthropic triatomine species widely distributed across southern China, carries *Rickettsia*, including *R. felis* and a *Rickettsia* sp. Using a six-gene multi-locus approach (*groEL*, *rrs*, *gltA*, *17-kDa*, *ompA*, and *ompB*), we detected *R. felis* and *Rickettsia* sp. in multiple tissues of *T. rubrofasciata* specimens collected from Guangxi and Hainan Provinces, including the head (presumably containing the salivary glands), gut, and fresh feces. These findings suggest that *T. rubrofasciata* may serve as a potential vector for *Rickettsia*, and point to triatomines as a novel candidate in the ecology of *Rickettsia*.

*R. felis*, a member of the spotted fever group (SFG), is an emerging human pathogen associated with fever, rash, headache, and severe complications such as encephalitis and pneumonia [41, 42]. First identified in the USA in 1994 [43], since then, human cases have been reported in at least 15 countries [44]. In China, the first confirmed case was documented in Jiangsu Province in 2014, and subsequent reports from Shandong, Zhejiang, and Shanxi provinces highlight its growing public health relevance [42, 45–47]. Despite increasing clinical recognition, the precise transmission dynamics of *R. felis* remain unclear. While the cat flea (*C. felis*) is the only arthropod with experimentally confirmed vector competence, *R. felis* DNA has been repeatedly detected in ticks, chiggers, booklice [48], and mosquitoes; however, biological transmission by these alternative hosts remains unproven.

Our concurrent detection of closely related *R. felis* strains in local *C. felis* and *R. norvegicus* strongly suggests an active enzootic transmission cycle involving domestic and peridomestic mammals in the study region. *C. felis* serves as the primary vector of *R. felis* and maintains a life cycle tightly linked to humans and domestic pets (cats and dogs); frequent blood-feeding by this flea facilitates cross-host transmission, increasing the risk of infection to humans and other animal hosts. *T. rubrofasciata,* which frequently feeds on rodents, dogs, cats, and humans in peridomestic settings [49], may acquire *R. felis* opportunistically during blood meals from infected hosts. Notably, the detection of *R. felis* DNA in the head (potentially salivary glands) and feces of triatomines mirrors the tissue tropism observed in *C. felis*, where the bacterium colonizes the midgut, salivary glands, and reproductive organs [50].

Nevertheless, we note that PCR positivity for *Rickettsia* could also result from the triatomine’s acquisition of the pathogen through a blood meal taken from an infected host. Several studies have shown that pathogens acquired by triatomines via a blood meal can persist for up to 2 weeks before disappearance of the pathogen in triatomines [51, 52]. Under such circumstances, the pathogen is unlikely to replicate within the triatomine, making it difficult to establish a new infection. Consequently, whether *T. rubrofasciata* can support *Rickettsia* replication and serve as a competent vector remains unknown and requires experimental verification. Nonetheless, the ecological overlap among fleas, rodents, and triatomines highlights the need for integrated surveillance of nontraditional vectors in the ecology of rickettsial diseases.

Multi-locus sequence analysis resolved three distinct rickettsial taxa in our samples: *R. felis* (in fleas, rodents, and triatomines), while an unconfirmed *Rickettsia* sp. closely related to *R. felis* was detected in low-yield triatomine and rodent samples, and *Candidatus* R. jingxinensis restricted to *R. microplus* ticks. Notably, all eight *T. rubrofasciata* specimens positive for *Rickettsia* belonged to the *R. felis* lineage, with four meeting the Fournier criteria for species-level assignment based on ≥ 99.8% identity across *rrs*, *gltA*, *ompA*, and *ompB* [35]. Phylogenetic analyses across all six loci consistently placed triatomine-derived sequences within the *R. felis* clade alongside flea and rodent isolates, while tick-derived sequences formed a separate, well-supported cluster with *Candidatus* R. jingxinensis. These findings delineate two distinct, yet spatially sympatric, transmission cycles: an *R. felis* cycle maintained between fleas and rodents, and a separate cycle involving ticks and *Candidatus* R. jingxinensis. Together, these overlapping cycles constitute an interconnected multi-host network, directly linking companion animals, wildlife, and humans within shared habitats.

Interestingly, sequence alignments of the *17-kDa*, *ompA*, and *ompB* genes across different vectors and hosts revealed that *ompA* and *ompB* sequences from triatomine and flea isolates were nearly identical to reference *R. felis* strains. In contrast, two rodent-derived isolates carried a distinct *ompA* haplotype characterized by 12 shared nucleotide substitutions. This divergence likely reflects either host-adapted evolution or the circulation of a murine-specific variant, highlighting the genetic plasticity and ecological complexity of *R. felis*.

Several limitations warrant consideration. First, the relatively small number of *T. rubrofasciata* specimens and limited geographic sampling may underestimate true prevalence. Second, and more critically, we cannot distinguish whether *R. felis* DNA in triatomines represents transient passage from an infected blood meal or genuine infection with potential for replication and transmission. The absence of histological or culture-based confirmation leaves vector competence unverified. Nevertheless, given that *T. rubrofasciata* exhibits feeding and defecation behaviors similar to those of *C. felis*—and frequently bites humans in homes and animal shelters—the theoretical risk of rickettsial transmission cannot be dismissed.

## Conclusion

This study documents the first detection of *Rickettsia* spp. in *T. rubrofasciata*, suggesting that this widespread synanthropic insect may serve as a previously unrecognized interface in the epidemiology of flea-borne spotted fever in southern China. Although experimental validation of transmission is still needed, the co-circulation of *R. felis* strains in fleas, rodents, and triatomines in the same locales suggests a complex, multi-host transmission network. Given the expanding human footprint in endemic areas and the increasing frequency of human-triatomine encounters, surveillance for rickettsial pathogens in nontraditional vectors such as triatomines should be prioritized. Future studies should (1) expand sampling across southern China to assess the geographic extent of *R. felis* in triatomines, and (2) conduct experimental infection studies to determine whether *R. felis* can replicate within *T. rubrofasciata* and be transmitted via its bite or feces-key steps toward evaluating its public health significance.

## Supplementary Information

Supplementary Material 1.

Supplementary Material 2.

Supplementary Material 3.

Supplementary Material 4.

Supplementary Material 5.

## Data Availability

All data supporting the findings of this study are available within the paper and its Supplementary Information. Sequence data were deposited into the Gene bank.
