# Supplementary material for: First molecular evidence of Rickettsia spp. in Triatoma rubrofasciata: implications for vector ecology and zoonotic transmission
Source: Parasit Vectors. 2026 Jun 13;19:272. doi: 10.1186/s13071-026-07489-9 (PMC13330430; doi:10.1186/s13071-026-07489-9)
Supplement: Supplementary file 1 — Supplementary Material 1. [file 13071_2026_7489_MOESM1_ESM.pdf]

Additional file 1: Table S1. Information on *17-kDa*, *ompA*, and *ompB* gene sequences of *Rickettsia felis* from various host origins retrieved from the NCBI database

***Rickettsia* spp.**	**Strain**	**Host**	**Country**	***17-kDa***	***ompB***	***ompA***
	flea41	*Ctenocephalides felis*	Malta	MK242056.1		
	AB3572	Homo sapiens	Thailand	OR567478.1		
	b22062	Homo sapiens	Japan	LC764839.1		
	CtML-23	cat flea	Malta	MG893577.1		
	LIC5552C	*Rhipicephalus microplus*	Brazil	MH194356.1		
	LIC 6132	*Amblyomma sculptum*	Brazil	KY273597.1		
	2018rom41	*Amblyomma geoemdae*	Taiwan		PQ151995.1	
	Sirumalai	*Haemaphysalis intermedia*	India		OM675973.1	
Rickettsia felis	93	*Rhiphicephalus sanguineus*	India		MN700900.1	
	GV669	*Questing Ixodes ricinus*	Brazil		MK301596.1	
	5396/17	dog	Italy		MG451836.1	
	Cl-Z4-2Rf	*Cimex lectularius*	Tunisia		PP330951.1	
	CAH46	*Amblyomma dissimile*	Colombia		PP820517.1	
	105_14	flea	Chile			KY913643.1
		Haemaphysalis intermedia	India			OM675977.1
	scc50	*Carios capensis*	USA			DQ102710.1
	LIS551C	*Canis familiaris*	Brazil			KY172883.1
