# Supplementary material for: First molecular evidence of Rickettsia spp. in Triatoma rubrofasciata: implications for vector ecology and zoonotic transmission
Source: Parasit Vectors. 2026 Jun 13;19:272. doi: 10.1186/s13071-026-07489-9 (PMC13330430; doi:10.1186/s13071-026-07489-9)
Supplement: Supplementary file 2 — Supplementary Material 2. [file 13071_2026_7489_MOESM2_ESM.pdf]

Additional file 2 : Table S2. Sequence information used for the phylogenetic reconstruction of *Rickettsia* spp.

***Rickettsia* spp.**	**Strain**	**Host**	**Country**	***groEL***	***rrs***	***gltA***	***17-kDa***	***ompA***	***ompB***
Rickettsia aeschlimannii	Baiyin-Ha-14	*Hyalomma asiaticum*	China	MH932043.1					
Rickettsia aeschlimannii	R102	tick	France			MK608596.1			
Rickettsia aeschlimannii	Stavropol-16	*Hyalomma marginatum*	Russia		ON721168.1				
Rickettsia africae	Do48F1	*Hyalomma dromedarii*	Tunisia					MN094830.1	
Rickettsia africae	Kankan-3/2024	*Amblyomma variegatum*	Guinea			PV261003.1			
Rickettsia africae	HuaAVRic5.4	*Amblyomma variegatum*	Portugal					MT085833.1	
Rickettsia amblyommatis	An13	*Amblyomma neumanni*	Argentina	CP015012.1				DQ517292.1	
Rickettsia amblyommatis	C33	chigger mites	USA				OM796109.1		
Rickettsia amblyommatis		tick	Eswatini			MZ351060.1			
Rickettsia asembonensis	PoFlRmt1	flea	Portugal						MK732016.1
Rickettsia asembonensis	RNMRS718	Flea	India				MN871513.1		
Rickettsia asembonensis	MUWRP-0062	*Rhipicephalus decoloratus*	Uganda				OP974448.1		
Rickettsia asiatica	NGT369-2017-Iova	*Ixodes ovatus*	Japan				LC461066.1		LC461081.1
Rickettsia asiatica	Iov_1277	*Ixodes ovatus*	Japan		LC379494.1	LC379441.1	LC379458.1		
Rickettsia conorii	MUWRP-0009	*Rhipicephalus decoloratus*	Uganda				OP974435.1		
Rickettsia conorii	Moroccan		Spain			PQ511235.1			
Rickettsia conorii subsp.	HY2	tick	China					OR944498.1	
Rickettsia conorii subsp.	BIME	*Dermacentor silvarum*	China	CP098324.1					
Rickettsia conorii subsp.	GV536	*Ixodes ricinus*	Spain						MK301599.1
Rickettsia conorii subsp. raoultii	HX-HY-17KDA-28	*Hyalomma*	China				MT795153.1		
Rickettsia endosymbiont	NIVEDI_2019_PK42	*Rhipicephalus microplus*	India					MN537556.1	
Rickettsia felis	Dog-470	dog	China		MT003287.1				
Rickettsia felis	Dog-8	dog	China	MT019639.1	MT003286.1	MT019627.1		MT019651.1	
Rickettsia felis	105_14	fleas	Chile					KY913643.1	
Rickettsia felis	0-OP-16-1	*Ctenocephalides felis*	Costa Rica					KX544814.1	
Rickettsia felis	2018rom41	*Amblyomma geoemdae*	Japan						PQ151995.1
Rickettsia felis	AB3572	Homo sapiens	Thailand				OR567478.1		
Rickettsia felis	28	*Ctenocephalides felis*	Austria					MF374382.1	
Rickettsia felis	Case_149	Homo sapiens	Viet Nam				OR640893.1		
Rickettsia felis	Case_174	Homo sapiens	Viet Nam				OR640894.1		
Rickettsia felis	Sirumalai	*Haemaphysalis intermedia*	India					OM675977.1	OM675973.1
Rickettsia felis	Vietnam 01	*Rhipicephalus sanguineus*	Vietnam			PP393500.1			
Rickettsia felis	scc50	*Carios capensis*	USA		DQ102712.1				
Rickettsia gravesii	BWI-1	*Amblyomma triguttatum*	Australia				DQ269436.1		
Rickettsia helvetica	Im-IZM	*Ixodes monospinosus*	Japan				LC334340.1		

Rickettsia helvetica	US002	Red deer	Spain						OP729875.1
Rickettsia helvetica	258	*Dermacentor reticulatus*	Russia			PV632059.1	GU827042.1		
Rickettsia helvetica	Svil2-6	*Ixodes ricinus*	Lithuania				MF491749.1		
Rickettsia hoogstraalii	Av202	*Argas transgariepinus*	South Africa				MH383145.1		
Rickettsia japonica	WHHFXZ-13	*Haemaphysalis flava*	China	KX987376.1	KX987313.1				
Rickettsia japonica		Homo sapiens	Japan				LC870168.1		
Rickettsia japonica	HS73	*Haemaphysalis flava*	South Korea					OL687166.1	
Rickettsia japonica	J83	*Haemaphysalis longicornis*	China		MF496163.1	MF496180.1		MK102718.1	
Rickettsia lusitaniae	OnP31/2019	*Ornithodoros porcinus*	Zambia				LC649693.1		
Rickettsia massiliae	GU037	*Ixodes ricinus*	Spain						ON859991.1
Rickettsia massiliae	18	*Rhipicephalus turanicus*	Lebanon				KY233204.1	KY233237.1	
Rickettsia massiliae	30gltA	tick	Portugal			PX438758.1			
Rickettsia monacensis	M61	*Ixodes ricinus*	Portugal			OR399958.1			
Rickettsia monacensis	GU034	*Rhipicephalus sanguineus*	Spain						ON859995.1
Rickettsia monacensis	KY088_Cl.6	nymph	Thailand			OR523981.1			
Rickettsia montanensis	OSU	*Cavia porcellus*	USA					MT035857.1	
Rickettsia parkeri	RparkeriAm24	*Amblyomma maculatum*	Ecuador						OR885265.1
Rickettsia parkeri	RPDPMEX	tick	Mexico			MG578512.1	MG578509.1	MG578510.1	
Rickettsia peacockii	CAA484	*Dermacentor andersoni*	Canada		HF935070.1	HF935076.1			
Rickettsia raoultii		tick	Khabarovsk	CP010969.1					
Rickettsia raoultii		Homo sapiens	Italy						KC847318.1
Rickettsia raoultii		*Haemaphysalis erinacei*	China		KR608783.1				
Rickettsia rhipicephali	RrMG	*Amblyomma sp.*	Brazil					KX018049.1	
Rickettsia rhipicephali	HJ5	*Haemaphysalis juxtakochi*	Brazil				DQ865207.1	DQ865208.1	
Rickettsia rhipicephali	ban2_DE_D90_PYG_39		China		MT903193.1				
Rickettsia rickettsii	Crystal Cove	*Dermacentor occidentalis*	USA	CP130462.1					
Rickettsia rickettsii	Colatina1	*Amblyomma cajennense nymph*	Brazil				KC845924.1	KC845925.1	
Rickettsia rickettsii	1991CO3	*Canis familiaris*	USA		DQ150685.1				
Rickettsia rickettsii str.	Iowa		USA	CP000766.3					
Rickettsia sibirica	O53	*Dermacentor silvarum*	China	CP170619.1					
Rickettsia sibirica subsp. mongolitimonae	GU079	*Ixodes frontalis*	Spain						ON859993.1
Rickettsia slovaca	E172	*Dermacentor marginatus*	Spain			MW835778.1			
Rickettsia slovaca	TPU051	*Hyalomma lusitanicum*	Spain						OP729870.1
Rickettsia tamurae	KY020_Cl.15	nymph	Thailand			OR523963.1			
Rickettsia typhi	2020-CO-Texas	*Canis lupus familiaris*	USA				OK073987.1		
Rickettsia typhi	F31	*Ctenocephalides felis*	USA			PP856021.1			
Rickettsia typhi	Wilmington		USA		L36221.1				
Rickettsia typhi	KRS13	*Ctenophthalmus congeneroides*	South Korea						HQ236390.1

Rickettsia vini	Boshoek1	*Ixodes arboricola*	Belgium		MT062904.1			MT062907.1	
Rickettsia vini	I.l.-LT	*Ixodes lividus*	Lithuania					MN078337.1	
Rickettsia sp.	Hainan 1		China	EU402925.1					
Candidatus *Rickettsia davousti*		*Amblyomma tholloni tick*	Gabon					DQ402517.1	
Candidatus *Rickettsia jingxinensis*	tick-XA188	*Haemaphysalis longicornis*	China	MH500219.1	MH500199.1				
Candidatus *Rickettsia jingxinensis*	Meixian-Hl-11	*Haemaphysalis longicornis*	China	MH932047.1	MH923220.1		MH932032.1	MH932062.1	
Candidatus *Rickettsia jingxinensis*	Xian-Hl-141	*Haemaphysalis longicornis*	China		MH923225.1		MH932037.1	MH932067.1	
Candidatus *Rickettsia jingxinensis*	TIGMIC-194	*Haemaphysalis longicornis*	China				PP117207.1	PP117714.1	
Candidatus *Rickettsia jingxinensis*	PT3-2	tick	China				PQ133370.1		
Candidatus *Rickettsia jingxinensis*	Patient_9	Homo sapiens	China						PP922939.1
Candidatus *Rickettsia jingxinensis*	Patient_1	Homo sapiens	China				PP922920.1		PP922931.1
Candidatus *Rickettsia jingxinensis*	2018rom88	*Amblyomma geoemdae*	Taiwan						PQ151996.1
Candidatus *Rickettsia jingxinensis*	TIGMIC-186	*Haemaphysalis longicornis*	China				PP117214.1	PP117694.1	
Candidatus *Rickettsia jingxinensis*	HBSJZJX40	*Haemaphysalis longicornis*	China			OQ702261.1			
Candidatus *Rickettsia jingxinensis*	Zhaotong-168	tick	China		OL855736.1	OL856122.1		OL856112.1	
Orientia tsutsugamushi	UT125	Homo sapiens	Thailand	EF551293.1				MH168006.1	
Orientia tsutsugamushi		Homo sapiens	Australia		AF062074.1				
