# Supplementary material for: First molecular evidence of Rickettsia spp. in Triatoma rubrofasciata: implications for vector ecology and zoonotic transmission
Source: Parasit Vectors. 2026 Jun 13;19:272. doi: 10.1186/s13071-026-07489-9 (PMC13330430; doi:10.1186/s13071-026-07489-9)
Supplement: Supplementary file 3 — Supplementary Material 3. [file 13071_2026_7489_MOESM3_ESM.pdf]

Additional file 3: Table S3. Detailed information on sample collection for triatomines, fleas, rodents, and ticks

Sample Species	Collection date	Collection site	Coordinates	Collection source	Collection method	Habitat type (Intra/Peri/Sylvatic)/Source	No. of specimens
triatomines	11 June 2023	Nanning,Xixiangtang	N/A	Resident-submitted	Manual turning and hand capture	Peri（Woodpiles near poultry shelters）	10
triatomines	25 June 2023	Laibin,Wuxuan	23.52°N, 109.60°E	Resident-submitted	Manual turning and hand capture	Peri（Debris piles under eaves）	8
triatomines	7 July 2023	Hezhou,Babu	N/A	Resident-submitted	Direct hand collection	Intra（Wooden bed boards）	3
triatomines	28 July 2023	Nanning,Qingxiu	N/A	Resident-submitted	Direct hand collection	Peri（Debris piles under eaves）	7
triatomines	5 August 2023	Nanning,Xixiangtang	22.84°N, 108.27°E	Researcher-collected	Direct hand collection	Peri（Woodpiles near poultry shelters）	14
triatomines	11 August 2023	Yulin,Bobai	N/A	Resident-submitted	Manual turning and hand capture	Peri（Woodpiles near poultry shelters）	9
triatomines	11 August 2023	Yulin,Xingye	N/A	Resident-submitted	Direct hand collection	Intra（Wooden bed boards）	2
triatomines	24 August 2023	Hechi,Nandan	N/A	Resident-submitted	Manual turning and hand capture	Peri（Woodpiles near poultry shelters）	16
triatomines	7 November 2023	Liuzhou,Liujiang	N/A	Resident-submitted	Manual turning and hand capture	Peri（Woodpiles near poultry shelters）	12
triatomines	18 May 2024	Chongzuo,Ningming	N/A	Resident-submitted	Manual turning and hand capture	Peri（Woodpiles near poultry shelters）	7
triatomines	19 June 2024	Laibin,Xingbin	N/A	Resident-submitted	Direct hand collection	Peri（Wooden window frames）	3
triatomines	21 July 2024	Qinzhou,Qinbei	22.19°N, 108.53°E	Resident-submitted	Manual turning and hand capture	Peri（Woodpiles near poultry shelters）	30
triatomines	23 July 2024	Baise,Pingguo	N/A	Resident-submitted	Direct hand collection	Intra（Wooden bed boards）	2
triatomines	11 August 2024	Nanning,Qingxiu	22.84°N, 108.42°E	Resident-submitted	Direct hand collection	Peri（Debris piles under eaves）	2
triatomines	15 August 2024	Baise,Youjiang	N/A	Resident-submitted	Manual turning and hand capture	Peri（Woodpiles near poultry shelters）	6
triatomines	25 August 2024	Nanning,Xixiangtang	22.84°N, 108.29°E	Resident-submitted	Direct hand collection	Intra（Wooden bed boards）	1
triatomines	15 September 2024	Beihai,Hepu	21.89°N, 109.64°E	Resident-submitted	Manual turning and hand capture	Peri（Woodpiles near poultry shelters）	20
triatomines	1 November 2024	Nanning,Xixiangtang	22.82°N, 108.29°E	Resident-submitted	Direct hand collection	Intra（Wooden bed boards）	1
triatomines	1 November 2024	Chongzuo,Jiangzhou	22.41°N, 107.37°E	Researcher-collected	Manual turning and hand capture	Peri（Woodpiles near poultry shelters）	28
triatomines	16 November 2024	Nanning,Xixiangtang	22.84°N, 108.33°E	Resident-submitted	Direct hand collection	Intra（Wall crevices inside houses）	1
triatomines	18 November 2024	Hainan,Lingao	19.91°N, 109.69°E	Resident-submitted	Manual turning and hand capture	Peri（Woodpiles near poultry shelters）	78
triatomines	2 February 2025	Qinzhou,Qinbei	22.19°N, 108.53°E	Resident-submitted	Manual turning and hand capture	Peri（Woodpiles near poultry shelters）	20
triatomines	3 May 2025	Qinzhou,Qinbei	22.19°N, 108.53°E	Resident-submitted	Manual turning and hand capture	Peri（Woodpiles near poultry shelters）	10
triatomines	9 May 2025	Hainan,Lingao	19.91°N, 109.69°E	Resident-submitted	Manual turning and hand capture	Peri（Woodpiles near poultry shelters）	34
triatomines	12 May 2025	Beihai,Hepu	21.89°N, 109.64°E	Resident-submitted	Manual turning and hand capture	Peri（Woodpiles near poultry shelters）	8
triatomines	13 May 2025	Laibin,Wuxuan	23.52°N, 109.60°E	Resident-submitted	Direct hand collection	Peri（Wooden window frames）	1
triatomines	9 July 2025	Qinzhou,Qinbei	22.19°N, 108.53°E	Resident-submitted	Direct hand collection	Intra（Wooden bed boards）	4
triatomines	30 July 2025	Chongzuo,Jiangzhou	22.41°N, 107.37°E	Researcher-collected	Manual turning and hand capture	Peri（Woodpiles near poultry shelters）	23
triatomines	15 August 2025	Beihai,Hepu	21.89°N, 109.64°E	Resident-submitted	Direct hand collection	Intra（Wooden bed boards）	1
triatomines	23 August 2025	Nanning,Qingxiu	22.81°N, 108.36°E	Resident-submitted	Direct hand collection	Intra（Wall crevices inside houses）	1
fleas	19 June 2024	Nanning,Xixiangtang	22.88°N, 108.29°E	Researcher-collected	Direct hand collection	cat	30

fleas	19 June 2024	Nanning,Xixiangtang	22.88°N, 108.29°E	Researcher-collected	Direct hand collection	dog	3
fleas	2 July 2024	Nanning,Xixiangtang	22.88°N, 108.29°E	Researcher-collected	Direct hand collection	cat	8
fleas	9 July 2024	Nanning,Xixiangtang	22.88°N, 108.29°E	Researcher-collected	Direct hand collection	cat	3
fleas	9 September 2024	Nanning,Xixiangtang	22.88°N, 108.29°E	Researcher-collected	Direct hand collection	cat	8
fleas	9 September 2024	Nanning,Xixiangtang	22.88°N, 108.29°E	Researcher-collected	Direct hand collection	dog	2
fleas	28 September 2024	Nanning,Xixiangtang	22.88°N, 108.29°E	Researcher-collected	Direct hand collection	cat	10
rats	31 January 2024	Hechi, Yizhou	N/A	Researcher-collected	live trapping	Peri (Sewage sewer)	30
rats	3 January 2025	Nanning, Qingxiu	22.81°N, 108.36°E	Researcher-collected	live trapping	Peri (Sewage sewer)	2
rats	27 August 2025	Nanning, Xixiangtang	22.92°N, 108.05°E	Researcher-collected	live trapping	Peri (Sewage sewer)	37
ticks	9 March 2023	Liuzhou,Rongshui	N/A	Resident-submitted	Direct hand collection	cattle	13
ticks	19 March 2023	Baise,Youjiang	N/A	Resident-submitted	Direct hand collection	cattle	20
ticks	19 March 2023	Baise,Youjiang	N/A	Resident-submitted	Direct hand collection	dog	6
ticks	24 July 2025	Hechi, Duan	23.92°N, 108.05°E	Resident-submitted	Direct hand collection	cattle	18
ticks	24 July 2025	Baise,Youjiang	N/A	Resident-submitted	Direct hand collection	cattle	20
ticks	24 July 2025	Baise,Youjiang	N/A	Resident-submitted	Resident-submitted	dog	4
ticks	24 July 2025	Hechi, Duan	23.96°N, 108.10°E	Resident-submitted	Resident-submitted	cattle	6
ticks	24 July 2025	Hechi, Duan	23.90°N, 108.28°E	Resident-submitted	Direct hand collection	cattle	9
ticks	24 July 2025	Hechi, Duan	23.98°N, 108.10°E	Resident-submitted	Direct hand collection	cattle	6
ticks	29 August 2025	Beihai,Hepu	21.85°N, 109.15°E	Resident-submitted	Direct hand collection	cattle	24
ticks	29 August 2025	Beihai,Haicheng	21.52°N, 109.17°E	Resident-submitted	Direct hand collection	dog	3
ticks	29 August 2025	Beihai,Weizhoudao	21.03°N, 109.12°E	Resident-submitted	Direct hand collection	cattle	2
ticks	19 October 2025	Nanning, Mashan	N/A	Resident-submitted	Direct hand collection	cattle	2
ticks	19 October 2025	Beihai,Hepu	21.85°N, 109.15°E	Resident-submitted	Direct hand collection	cattle	3

N/A, not available.
