# Supplementary material for: First molecular evidence of Rickettsia spp. in Triatoma rubrofasciata: implications for vector ecology and zoonotic transmission
Source: Parasit Vectors. 2026 Jun 13;19:272. doi: 10.1186/s13071-026-07489-9 (PMC13330430; doi:10.1186/s13071-026-07489-9)
Supplement: Supplementary file 4 — Supplementary Material 4. [file 13071_2026_7489_MOESM4_ESM.pdf]

Additional file 4: Table S4. Detailed information on PCR amplification and species identification of *Rickettsia* from *Triatoma rubrofasciata*, *Ctenocephalides felis*, *Rattus norvegicus*, and *Rhipicephalus microplus*

Species	Sample ID	Location	Host / Tissue	Closest similarity in GenBank according to rickettsial genes (accession numbers in parentheses)					
				groEL	***rrs***	***gltA***	***17kDa***	***ompA***	***ompB***
Triatoma rubrofasciata	GxTrRi-4	Qinzhou,Qinbei	heads/gut/feces	*R. felis* (MT019639.1) 98.87%	Not successful	Not successful	*R.felis* (MK242042.1)99.73%	*R.felis* (KY913641.1)99.56%	Not successful
Triatoma rubrofasciata	GxTrRi-10	Nanning,Qingxiu	heads/feces	*R.felis* (MT019639.1)98.94%	Not successful	Not successful	Not successful	Not successful	Not successful
Triatoma rubrofasciata	GxTrRi-11	Qinzhou,Qinbei	heads/gut/feces	*R. felis* (MT019639.1)99.44%	Not successful	Not successful	Not successful	Not successful	Not successful
Triatoma rubrofasciata	GxTrRi-24	Qinzhou,Qinbei	gut/feces	*R. felis* (MT019639.1)98.88%	*R.felis* (MT003287.1)99.88%	*R.felis* (KF242471.1) 100%	*R.felis* (MK242042.1)99.73%	Not successful	*R.felis* (PP820517.1)99.21%
Triatoma rubrofasciata	GxTrRi-26	Qinzhou,Qinbei	gut/feces	*R. felis* (MT019639.1)99.44%	Not successful	Not successful	Not successful	Not successful	Not successful
Triatoma rubrofasciata	GxTrRi-57	Chongzuo,Jiangzhou	gut/feces	*R. felis* (MT019639.1)98.95%	Not successful	Not successful	Not successful	*R.felis* (KY913646.1)99.57%	*R.felis* (PQ151995.1)100%
Triatoma rubrofasciata	GxTrRi-87	Chongzuo, Jiangzhou	gut/feces	*R.felis* (MT019639.1)98.88%	Not successful	Not successful	Not successful	Not successful	Not successful
Triatoma rubrofasciata	HnTrRi-5	Hainan,Lingao	feces	*R. felis* (MT019639.1)99.42%	*R.felis* (MT003287.1)99.88%	*R.felis* (KF242471.1) 100%	*R. felis* (MK242042.1)100%	*R. felis* (KY913639.1)100%	*R.felis* (PP820517.1)99.21%
Ctenocephalides felis	GxCfRi-2	Nanning, Xixiangtang	dog	*R.felis* (MT019639.1)96.28%	*R.felis* (MT003287.1)99.88%	*R.felis* (MW864276.1)100%	*R.felis* (OR567478.1)100%	*R.felis* (MT036381.1)100%	*R.felis* (OM675973.1)100%
Ctenocephalides felis	GxCfRi-3	Nanning, Xixiangtang	cat	*R.felis* (MT019639.1)96.69%	*R.felis* (MT003287.1)99.77%	*R.felis* (MW864276.1)100%	*R. felis* (MK242042.1)100%	*R.felis* (MT036381.1)100%	*R.felis* (PQ151995.1)100%
Ctenocephalides felis	GxCfRi-12	Nanning, Xixiangtang	dog	*R.felis* (MT019639.1)98.31%	*R.felis* (MT003287.1)99.77%	*R.felis* (MW864276.1)100%	*R. felis* (OR567478.1)100%	*R.felis* (MT036381.1)100%	*R.felis* (OM675973.1)100%
Ctenocephalides felis	GxCfRi-13	Nanning, Xixiangtang	cat	*R.felis* (MT019639.1)99.45%	R.felis (DQ102712.1)99.88%	*R.felis* (MW864276.1)100%	*R.felis* (OR567478.1)100%	*R.felis* (MT036381.1)100%	*R.felis* (OM675973.1)100%
Ctenocephalides felis	GxCfRi-28	Nanning, Xixiangtang	cat	*R. felis* (MT019639.1)99.45%	*R.felis* (MT003287.1)99.88%	*R.felis* (MW864276.1)100%	*R.felis* (OR567478.1)100%	*R.felis* (MT036381.1)100%	*R.felis* (PQ151995.1)100%
Ctenocephalides felis	GxCfRi-34	Nanning, Xixiangtang	cat	*R.felis* (MT019639.1)99.44%	*R.felis* (MT003287.1)99.77%	*R.felis* (MW864276.1)100%	*R.felis* (MF175774.1)100%	*R.felis* (MT036381.1)100%	*R.felis* (OM675973.1)100%
Ctenocephalides felis	GxCfRi-36	Nanning, Xixiangtang	cat	*R.felis* (MT019639.1)98.31%	*R.felis* (MT003287.1)99.42%	*R.felis* (MW864276.1)100%	*R.felis* (OR567478.1)100%	*R.felis* (MT036381.1)100%	*R.felis* (PQ151995.1)100%
Ctenocephalides felis	GxCfRi-39	Nanning, Xixiangtang	cat	*R.felis* (MT019639.1)99.44%	*R.felis* (MT003287.1)99.88%	*R.felis* (MW864276.1)100%	*R.felis* (OR567478.1)100%	*R. felis* (MT036381.1)100%	*R.felis* (PQ151995.1)100%
Ctenocephalides felis	GxCfRi-44	Nanning, Xixiangtang	cat	*R.felis* (MT019639.1)99.44%	*R.felis* (MT003287.1)99.53%	*R.felis* (MW864276.1)100%	*R.felis* (OR567478.1)100%	*R.felis* (MT036381.1)100%	*R.felis* (OM675973.1)100%
Ctenocephalides felis	GxCfRi-52	Nanning, Xixiangtang	cat	*R.felis* (MT019639.1)98.87%	Not successful	*R.felis* (MW864276.1)100%	*R.felis* (OR567478.1)100%	*R.felis* (MT036381.1)100%	*R.felis* (PQ151995.1)100%
Rattus norvegicus	GxRaRi-33	Nanning, Xixiangtang	heart	Not successful	Not successful	Not successful	*R.felis* (OR567478.1)99.73%	*R.felis* (OR083232.1)97.48%	Not successful
Rattus norvegicus	GxRaRi-36	Nanning, Xixiangtang	heart	Not successful	*R.felis* (MT003287.1)99.73%	Not successful	*R.felis* (OR567478.1)100%	*R.felis* (KM006812.1)97.95%	Not successful
Rattus norvegicus	GxRaRi-66	Nanning, Xixiangtang	heart	Not successful	*R.felis* (MT003287.1)99.42%	*R.felis* (MW864275.1)100%	*R.felis* (OR567478.1)99.73%	Not successful	*R.felis* (PQ151995.1)100%
Rhipicephalus microplus	GxPcRi-7	Liuzhou, Rongshui	cattle	*Rickettsia* sp. (GQ499936.1)100%	Candidatus *R. jingxinensis* (OP764497.1)99.77%	Candidatus *R. jingxinensis* (OP080659.1)99.84%	Candidatus *R. jingxinensis* (PP117206.1)99.48%	Candidatus *R. jingxinensis* (OP776196.1)99.59%	Candidatus *R. jingxinensis* (PP922937.1)100%
Rhipicephalus microplus	GxPcRi-9	Liuzhou, Rongshui	cattle	*Rickettsia* sp. (GQ499936.1)99.43%	Candidatus *R. jingxinensis* (OP047983.1)100%	Candidatus *R. jingxinensis* (OQ702262.1)99.84%	Candidatus *R. jingxinensis* (OQ581084.1)99.74%	Candidatus *R. jingxinensis* (OP776196.1)99.59%	Candidatus *R. jingxinensis* (PQ151996.1)99.37%

Rhipicephalus microplus	GxPcRi-13	Liuzhou, Rongshui	cattle	*Rickettsia* sp. (GQ499936.1)96.11%	Candidatus *R. jingxinensis* (MH923219.1)99.88%	Candidatus *R. jingxinensis* (OP080659.1)99.84%	Candidatus *R. jingxinensis* (OQ702257.1)100%	Candidatus *R. jingxinensis* (OP776196.1)99.59%	Candidatus *R. jingxinensis* (PP922937.1)100%
Rhipicephalus microplus	GxPcRi-60	Baise,Youjiang	cattle	*Rickettsia* sp. (GQ499936.1)100%	Candidatus *R. jingxinensis* (OP047983.1)100%	Candidatus *R. jingxinensis* (OP080659.1)99.68%	Candidatus *R. jingxinensis* (PP117206.1)99.48%	Candidatus *R. jingxinensis* (OP776196.1)99.60%	Candidatus *R. jingxinensis* (PP922937.1)99.71%
Rhipicephalus microplus	GxPcRi-67	Baise,Youjiang	cattle	*Rickettsia* sp. (GQ499936.1)100%	Candidatus *R. jingxinensis* (MH923219.1)99.88%	Candidatus *R. jingxinensis* (OQ702261.1)99.52%	Candidatus *R. jingxinensis* (PQ133377.1)100%	Candidatus *R. jingxinensis* (OP776196.1)99.40%	Candidatus *R. jingxinensis* (PP922937.1)99.42%
Rhipicephalus microplus	GxPcRi-68	Baise,Youjiang	dog	*Rickettsia* sp. (EU402927.1)100%	Candidatus *R. jingxinensis* (OP047983.1)100%	Candidatus *R. jingxinensis* (OQ702261.1)99.84%	Candidatus *R. jingxinensis* (OQ581084.1)99.74%	Candidatus *R. jingxinensis* (OP776196.1)99.60%	Candidatus *R. jingxinensis* (PP922937.1)100%
Rhipicephalus microplus	GxPcRi-81	Baise,Youjiang	dog	*Rickettsia* sp. (GQ499936.1)100%	Candidatus *R. jingxinensis* (OR922297.1)99.88%	Candidatus *R. jingxinensis* (OQ702262.1)99.84%	Candidatus *R. jingxinensis* (OQ581084.1)99.74%	Candidatus *R. jingxinensis* (OP776196.1)99.60%	Candidatus *R. jingxinensis* (PP922937.1)100%
Rhipicephalus microplus	GxPcRi-90	Hechi, Duan	cattle	*Rickettsia* sp. (GQ499936.1)100%	Candidatus *R. jingxinensis* (OR922297.1)99.88%	Candidatus *R. jingxinensis* (OQ702261.1)99.84%	Candidatus *R. jingxinensis* (PQ133377.1)100%	Candidatus *R. jingxinensis* (OP296526.1)100%	Candidatus *R. jingxinensis* (ON600653.1)99.46%
Rhipicephalus microplus	GxPcRi-94	Hechi, Duan	cattle	*Rickettsia* sp. (GQ499936.1)100%	Candidatus *R. jingxinensis* (OP047983.1)100%	Candidatus *R. jingxinensis* (OP080659.1)99.36%	Candidatus *R. jingxinensis* (OQ581084.1)99.74%	Candidatus *R. jingxinensis* (OP296526.1)100%	Candidatus *R. jingxinensis* (ON600653.1)99.46%
Rhipicephalus microplus	GxPcRi-127	Beihai,haicheng	cattle	*Rickettsia* sp. (GQ499941.1)100%	Candidatus *R. jingxinensis* (OP047983.1)100%	Candidatus *R. jingxinensis* (OP080659.1)99.52%	Candidatus *R. jingxinensis* (PP117210.1)99.48%	Candidatus *R. jingxinensis* (OL856107.1)99.78%	Candidatus *R. jingxinensis* (PP922932.1)99.38%
Rhipicephalus microplus	GxPcRi-136	Beihai, hepu	cattle	*Rickettsia* sp. (GQ499954.1)100%	Candidatus *R. jingxinensis* (MH923219.1)99.88%	Candidatus *R. jingxinensis* (OQ702262.1)99.68%	Candidatus *R. jingxinensis* (PP117210.1)99.48%	Candidatus *R. jingxinensis* (PP116501.1)99.78%	Not successful

Not successful, PCR amplification failed.
