# Supplementary material for: First molecular evidence of Rickettsia spp. in Triatoma rubrofasciata: implications for vector ecology and zoonotic transmission
Source: Parasit Vectors. 2026 Jun 13;19:272. doi: 10.1186/s13071-026-07489-9 (PMC13330430; doi:10.1186/s13071-026-07489-9)
Supplement: Supplementary file 5 — Supplementary Material 5. [file 13071_2026_7489_MOESM5_ESM.pdf]

Supplementary Information

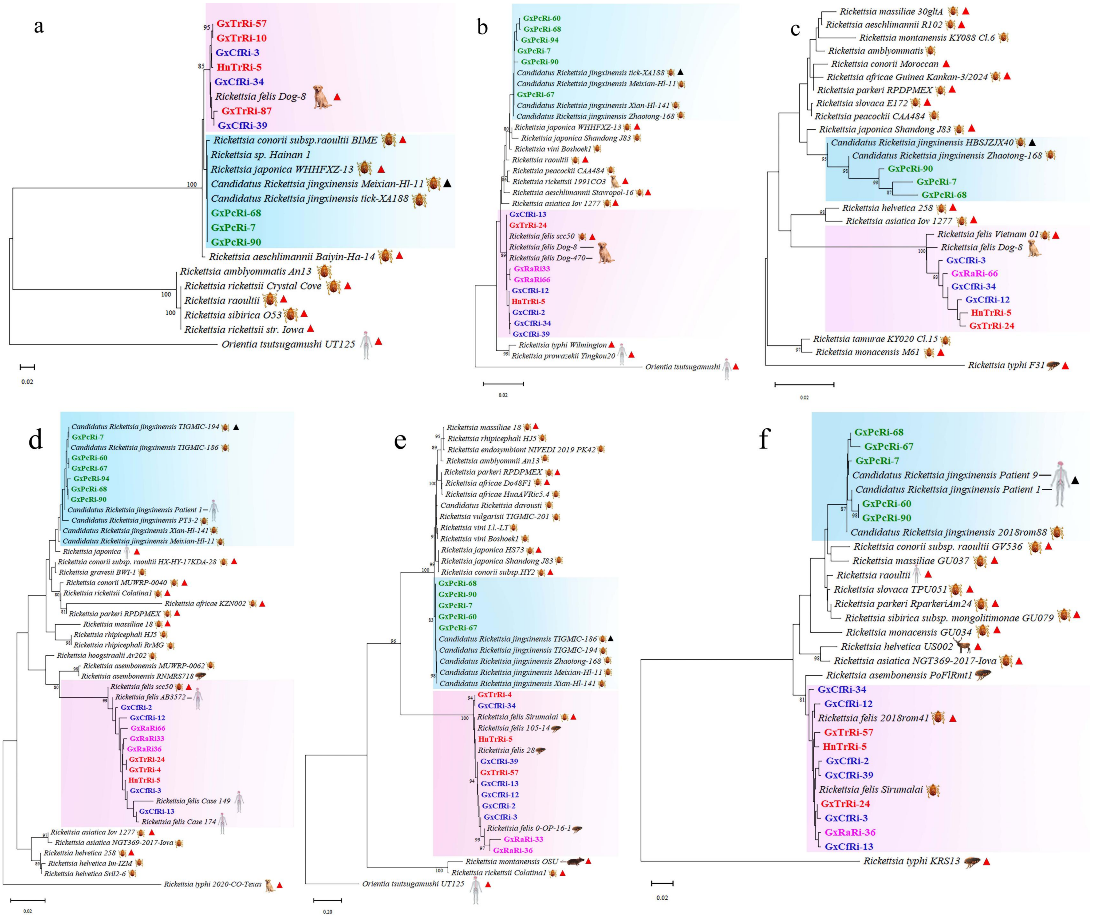

Fig. S1 Phylogenetic trees of *Rickettsia* spp. inferred from the *groEL* (a), *rrs* (b), *gltA* (c), *17-kDa* (d), *ompA* (e), and *ompB* (f) gene sequences.

Sequences generated in this study from *Triatoma rubrofasciata*, *Ctenocephalides felis, Rattus norvegicus,* and *Rhipicephalus microplus* are highlighted in red, blue, purple, and green fonts, respectively; corresponding GenBank accession numbers are listed in Additional file 2: Table S2. Host species of reference sequences are denoted by pictograms. Rickettsia taxa documented as human pathogens are indicated by red triangles, whereas those detected in humans but of unresolved pathogenicity are marked with black triangles. Trees were constructed using the neighbor-joining algorithm in MEGA11 with 1,000 bootstrap replicates.
